# Induction of Cardiac Pathology: Endogenous versus Exogenous Nrf2 Upregulation

**DOI:** 10.3390/cells11233855

**Published:** 2022-11-30

**Authors:** Bryan J. Mathis, Hideyuki Kato, Yuji Hiramatsu

**Affiliations:** 1 International Medical Center, University of Tsukuba Hospital, Tsukuba 305-8576, Ibaraki, Japan; 2 Department of Cardiovascular Surgery, Faculty of Medicine, University of Tsukuba, Tsukuba 305-8575, Ibaraki, Japan

**Keywords:** Nrf2, Keap1, cardiomyopathy, bardoloxone, Reata, CDDO

## Abstract

Nuclear factor erythroid 2-related factor 2 (Nrf2) is a master regulator of the endogenous antioxidant response to reactive oxygen species as well as a controller of Phase II detoxification in response to xenobiotics. This amenity to specific external manipulation exploits the binding affinity of Nrf2 for its constitutive repressor and degradation facilitator Kelch-like erythroid cell-derived protein with CNC homology-associated protein 1 (Keap1). Derived from both natural and synthesized origins, these compounds have been extensively tested without definitive beneficial results. Unfortunately, multiple terminated trials have shown a negative side to Nrf2 with regard to cardiac pathologies while animal-based studies have demonstrated cardiomyocyte hypertrophy and heart failure after chronic Nrf2 upregulation. Putatively based on autophagic control of Nrf2 activity-modulating upstream factors, new evidence of miRNA involvement has added complexity to this mechanism. What follows is an extensive survey of Nrf2-regulating exogenous compounds that may promote cardiomyopathy, clinical trial evidence, and a comparison to exercise-induced factors that also upregulate Nrf2 while preventing cardiac pathologies.

## 1. Introduction

Reactive oxygen species (ROS) are both a normal byproduct of mitochondrial metabolism and an endproduct of oxidative biochemical reactions in the cell. Balanced levels of subcellular compartmental ROS are important for normal cellular functions, whereas dysregulated ROS, usually caused by relative insufficiency or impairment of the endogenous antioxidant defense system, attack cellular components leading to cellular damage and death, a state referred as to oxidative stress. To maintain cellular redox homeostasis and neutralize uncontrolled ROS, conserved antioxidant defense enzymes are placed under the control of the nuclear factor erythroid 2-related factor 2 (Nrf2) gene which is constitutively expressed in all higher-order animals. This gene, as a master antioxidant transcription factor, is responsible for global antioxidant activity in response to internally and externally sourced ROS threats but also modulates such species to maintain important intracellular second messenger capability. As the inhibitor of Nrf2, Kelch-like erythroid cell-derived protein with CNC homology-associated protein 1 (Keap1), is amenable to attack by exogenous compounds, research has focused its efforts to increase Nrf2 activity via direct interaction with Keap1. These compounds, such as oleanic acid derivative 2-cyano-3,12-dioxoolean-1,9- dien-28-oic acid (CDDO, bardoloxone), have been intensely studied in animal models and human trials as potential defense agents oxidative stress-associated diseases, such as cancer, chronic kidney disease, fatty liver, and endometriosis. Unfortunately, human trials employing CDDO and similar compounds for amelioration of these maladies have met with ambiguous and often disappointing results. Furthermore, multiple trials throughout 2007–2014 were terminated as unforeseen myocardial pathologies resulted. What follows is a survey of Nrf2, its endogenous regulation, action, and potential for exogenous modulation. Additionally, current clinical trial results are presented and analyzed for characteristics of Nrf2 upregulation that result in possible cardiac issues. Finally, comparisons between these pharmaceutical regulators and exercise are made from mechanistic and physiological viewpoints to elucidate the differences between endogenous and exogenous regulatory effects on Nrf2 and cardiac health.

## 2. Nrf2 Composition

Nrf2 is a basic leucine zipper (bZIP) transcription factor on chromosome 2 in humans, comprised of a common, conserved Cap ‘n’ Collar (CNC) motif of 43 amino acids close to the DNA binding domain [1]. NRF2 consists of 6 exons, encoding 7 Nrf2-ECH homology (Neh) domains, and generates a 67.8 kDa protein from a 605 aa sequence and 2859 bp mRNA strand [2,3,4]. These Neh domains are specific for protein–protein interactions, especially regulatory, degradation, and translocation proteins (Table 1). The half-life of Nrf2 in the cytosol may be as little as 10 min if redox homeostasis is present or as long as 40 min under oxidative stress, relying on a sensitive Neh2-ETGE hinge region and redox-insensitive (but GSK3 interacting) Neh6 region to modulate binding to Keap1 and ubiquitin ligases [5,6]. Nrf2 Neh regions have been extensively illustrated, reviewed, and mapped but Nrf2 crystalline structure without Keap1 binding is sparse, indicating the importance of Keap1 binding in Nrf2 conformation [7,8]. Diverse variants of Nrf2 have been discovered but 9 are predicted to mediate a disease process and 8 mutagenic variants experience either loss of Keap1 binding or function (Table 2) [7].

### 2.1. Nrf2 Activation Mechanism

The Nrf2 activation mechanism has been canonically divided into 4 stages, highlighted by interactions with ROS-sensitive regulatory elements and translocation machinery. These stages are basal expression/repression, pre-induction, full induction, and post-induction [8].

#### 2.1.1. Basal Expression/Repression of Nrf2: Keap1, ROS, and Autophagy

Like the rest of the CNC family of transcription factors, Nrf2 is activated under stress, namely oxidative stress, and is related to a family of similar stress-response factors (e.g., Nrf1) [1]. Exercise, especially aerobic exercise, is also a potent inducer of Nrf2 (see Section 4.1). It is constitutively expressed and maintains its own 9nt upstream ARE sequence [13]. Levels of Nrf2 are tightly controlled by RONS-sensitive Keap1 through its modulation of K48-linked ubiquitination, together with backup systems such as β-TrCP and Hrd1 (see below).

Keap1 is a 70 kDa protein with a long 12.7 h half-life that localizes to the cytoplasm [7]. It is comprised of a BTB (Broad, Tramtrack and Bric-a-brac region) domain and Kelch repeats that bind directly to Nrf2-Neh2 in a 6-blade, *β*-propeller configuration that permits dimerization of 2 Keap1 molecules to each Nrf2 molecule in a hinged-capture structure with ETGE and DLG regions on the C-terminal end of Nrf2 to act as pivoting attachment points to Keap1 [4,7]. Under basal conditions, the conformational change induced by the Keap1-Nrf2 complex, NEDD8, and ubiquitin E3 ligase CUL3 exposes lysine residues within Neh2 (and possibly Neh6) to attack by a K48 polyubiquitination complex consisting of CUL3 and ring ligase RBX1 that bind to the BTB region of Keap1 before activation [7,14]. Subsequent proteasomal degradation of K48-polyubiquitinated Nrf2 then occurs within the cytoplasm. The binding affinity of Kesp1 to Nrf2 has been experimentally reported as KD 20 nM and, as such, spontaneous dissociation is unlikely [15]. However, 27 cysteine residues of Keap1 are vulnerable to attack by endogenous and exogenous reactive species, particularly C151 in the homodimerizing BTB region, that dissociate the CUL3 ubiquitin adaptor from the complex, allowing Nrf2 to escape polyubiquitination and begin translocation to the nucleus [4,16,17]. The p62/mTORC-1 dependent machinery, activated by autophagy, can also repress Keap1 by degrading it in the autophagic pathway [18,19]. Other p62-associated molecules, such as TFEB, can also protect Nrf2 by reducing ubiquitination through suppression of E3 ligase complex members (DACF11) while upregulating p62 to inhibit Keap1 binding to Nrf2 [20]. Of note, Nrf2 can also be repressed in a Keap1-independent manner by β-TrCP, which binds to Nrf2-Neh6 in a GSK-3 phosphorylation-dependent manner to facilitate SKP1-CUL1-RBX1/ROC1 ubiquitination [19,21]. E3 ubiquitin protein ligase HRD1 is also involved in Keap1 independent Nrf2 degradation [6]. Constitutively expressed proteins that generate ROS, such as NADPH oxidase-4 (NOX4) are also important in activating Nrf2 translocation [22].

Recent evidence has shown miRNA involvement in post-transcriptional regulation of Nrf2, with miRs -144, -28, -34, and -93 (among others) shown to decrease Nrf2 activity in animal models, while HuR and AUF1 RNA-binding proteins contribute to export and stabilization of the Nrf2 mRNA [3]. Constitutive expression and a short half-life, coupled with exquisitely sensitive, cysteine-based ROS sensors on Keap1 and links to autophagy and GSK-3 pathways, give Nrf2 the speed to react and fluctuate to maintain redox homeostasis under changing conditions.

#### 2.1.2. Nrf2 Action: Pre-Induction

After release, PI3K phosphorylation and Importin a5/B1 binding to specific nuclear localization signals on Nrf2 C- and N-terminal regions (Neh2 aa 42-53 and Neh3 aa 587-593) occurs to facilitate nuclear entry [23,24]. AMPK aids in nuclear accumulation by phosphorylating Ser558 to prevent export [24]. Nrf2 then begins to complex with Maf family members, Creb, and p300 adaptors to form a transcription-initiating complex [10].

#### 2.1.3. Nrf2 Full Induction, Transcription, and Purpose of Target Genes

Once translocation and complex formation are complete, the Nrf2-Maf-Cred-p300 complex binds a wide spectrum of antioxidant response elements (ARE), located 40 to 200 nucleotides upstream of transcription start sites, that encode Phase II detoxification, antioxidant enzyme, energy metabolism, and diverse other genes [13]. These short (9nt) sequences vary by gene and have been extensively reviewed by Raghunath et al. [13]. The Nrf2-Maf-Cred-p300 complex has been reported to control transcription of over 1000 genes and Table 3 shows a selection of genes related to the antioxidant and proliferation responses [25,26]. Of importance are catalases, glutathione S-transferases and cysteine-rich thioredoxins that detoxify xenobiotics, proteins with disulfide bonds, and ingested toxins, as well as ROS from mitochondrial respiration [27].

Of note, Bach1, which competitively binds with ARE sequences in concert with small Maf molecules, has emerged as an important modulator of Nrf2 transcriptional activity since it can directly interact to sense heme and act as a feedback inhibitor for promotion of HO-1 and NQO1 [28,29]. Bach1 is a member of the same CNC family as Nrf2 and is involved in induction of iron-induced immune cell apoptosis (ferroptosis) through prevention of antioxidant genes that counter iron-induced ROS [30].

Not limited to antioxidant defense alone, Nrf2 controls genes from multiple pathways as seen in the recent discovery of Nrf2-mediated cardiac hypertrophy from exogenous upregulators. Nrf2 controls cellular proliferation through PHGDH, PSAT1, SHMT1 and other ser/gly synthesis genes via interaction with ATF4 [25,31,32]. It additionally maintains a favorable redox status to facilitate mRNA translation, upregulates glycolysis/energy metabolism, and also contributes to stem cell viability through ROS regulation plus NOTCH and SIRT1 expression [3,33,34,35,36,37,38]. Thus, Nrf2 is an important co-initiator of the proliferative machinery, energy production, and facilitative redox control needed to prevent ROS damage from increased cellular growth and proliferation. It is these non-ARE effects that may be responsible for the cardiac maladaptation and hypertrophy seen in studies of exogenous Nrf2 upregulators.

**Table 3:** Select Genes Controlled by Nrf2 [39].

Function	Gene	Description	Ref.
Detoxification Phase II	*AHR*	Aryl hydrocarbon receptor	[2]
	*CYP1B1*	Cytochrome P450 Family 1 Subfamily B Member 1	[2]
	*ALDH3A2*	Aldehyde Dehydrogenase 3 Family Member A2	[2]
	*NQO1*	NAD(P)H Quinone Dehydrogenase 1	[2]
	*AKR1C1*	Aldo-Keto Reductase Family 1 Member C1	[40]
	*GSTM3*	Glutathione S-Transferase Mu 3	[40]
Antioxidant Defense	*GPX4*	Glutathione Peroxidase 4	[2]
	*GSR1*	Glutathione reductase, mitochondrial	[2]
	*TXN1*	Thioredoxin	[2]
	*PRDX1*	Peroxiredoxin 1	[2]
	*SRXN1*	Sulfiredoxin 1	[2]
	*SOD1/2*	Superoxide dismutase 1 and 2	[41]
	*HO-1*	Heme Oxygenase 1	[42]
	*GSTM3*	Glutathione S-Transferase Mu 3	[40]
Pentose Phosphate Pathway	*G6PD*	Glucose-6-Phosphate Dehydrogenase	[40]
	*PGD*	Phosphogluconate dehydrogenase	[3]
	*TKT*	Transketolase	[3]
Serine/Glycine Biosynthesis	*PHGDH*	Phosphoglycerate Dehydrogenase	[31]
	*PSAT1*	Phosphoserine Aminotransferase 1	[31]
	*SHMT1/2*	Serine Hydroxymethyltransferase 1/2	[31]
Membrane Trafficking	*RAB6B*	Ras-related protein Rab-6B	[40]
Deubiquitination	*UCH-L1*	Ubiquitin C-terminal hydrolase L1	[40]
Zinc Finger Protein	*TRIM16L*	Tripartite motif-containing protein 16	[40]
Glycolysis/ Glycogen Synthesis	*HK1/2*	Hexokinase 1 and 2	[3]
	*GP11*	Glucose phosphate isomerase 1	[3]
	*ALDA*	Fructose-bisphosphate aldolase A	[3]
	*ENO1*	Enolase 1	[3]
	*PKM2*	Pyruvate kinase muscle isoform 2	[3]
	*GLUT1*	Glucose transporter 1	[3]

#### 2.1.4. Nrf2 Post-Induction: Proteasomal Degradation

The 4–5 h window for Nrf2 transcriptional promotion is tightly controlled by phosphorylation, as GSK3 can phosphorylate Nrf2 to reduce its activity and kinases (Fyn and MAPK) prepare Nrf2 for nuclear export [24]. Fyn kinase interacts specifically with Tyr568 on Nrf2 to prepare it for export and another study by Li et al. has also found a leucine-enriched sequence (537-LKKQLSTLYL-546) resident in the Nrf2-Neh1 region that aids in CRM1 interaction for nuclear export [43]. A Neh6 region, containing a GSK3-interacting domain, was reported by McMahon et al. to promote destabilization of Nrf2 in a redox-insensitive manner [5]).

Once exported, the ubiquitin-proteasome complex is free to bind with Nrf2 and degrade it. Recent evidence has also hinted that the nucleus may play a role in degradation through the involvement of promyelocytic leukemia-nuclear bodies (PML-NB), comprised of PML and Sp100 proteins, in a process that sumoylates Nrf2 to render it susceptible to SUMO-targeting ubiquitin ligases [44]. Such regulation has been found to occur at 532-LKDE-535 and putatively at Lys100 (in mice), functioning to stabilize Nrf2 within the nucleus [45]. Thus, multiple degradation domains and pathways, with both redox-sensitive and -insensitive activity, ensures that Nrf2 can be quickly targeted for recycling to maintain rapid turnover and tight control of intracellular Nrf2 protein levels.

## 3. Effects of Nrf2 in the Heart versus Other Systems

The ability to engage a panoply of antioxidant and pro-growth factors upon ROS challenge, whether from endogenous sources or xenobiotics, makes Nrf2 highly desirable for manipulation to prevent oxidative damage. However, exogenous upregulation of Nrf2 beyond the control of repressive/degradation machinery may be deleterious as seen in studies linking upregulation of Nrf2 to cardiac hypertrophy and immune evasion/chemotherapy resistance in cancers [46,47]. What follows is a brief survey of the role of Nrf2 in the heart with a comparison to the kidneys to evaluate any potential side effects of exogenous Nrf2 enhancers.

### 3.1. Nrf2 in the Myocardium: Not a Silver Bullet

The heart is obligately aerobic and relies on oxidative phosphorylation to generate the biochemical energy needed for a lifetime of pumping. The coronary arteries supply oxygenated blood to the heart during diastole and increases in oxygen demand by the myocardium are directly related to the heart rate (higher rate = higher oxygen demand and shorter diastole for coronary supply) and saturation of blood by oxygen (to prevent hypoxia). Even at rest, the myocardium consumes 8 to 13 mL of oxygen per 100 g of tissue per minute and ROS from mitochondrial respiration and pro-ROS proteins, such as Nox4, create a pro-oxidative state that requires constant rebalancing to maintain redox homeostasis [48,49]). Xenobiotics may also introduce ROS either by direct chemical action (e.g., nitrosamines from cigarette smoke, fermented foods, or cured meats) or immune response. However, since ROS function as a second-messenger system and have been implicated as crucial regulators of stem cell differentiation and apoptosis/necrosis, tight regulation of the Nrf2-mediated antioxidant response (e.g., via Keap1 direct and Bach1 competitive pathways) is required to maintain such basal messenger activity. Cardiomyocyte differentiation, in particular, is sensitive to ROS, requiring it for progression to maturity, and cardiac-resident stem cells in adults may be similarly affected by imbalanced redox homeostasis, driving them towards hypertrophic or synthetic phenotypes [50].

**Figure 1:**
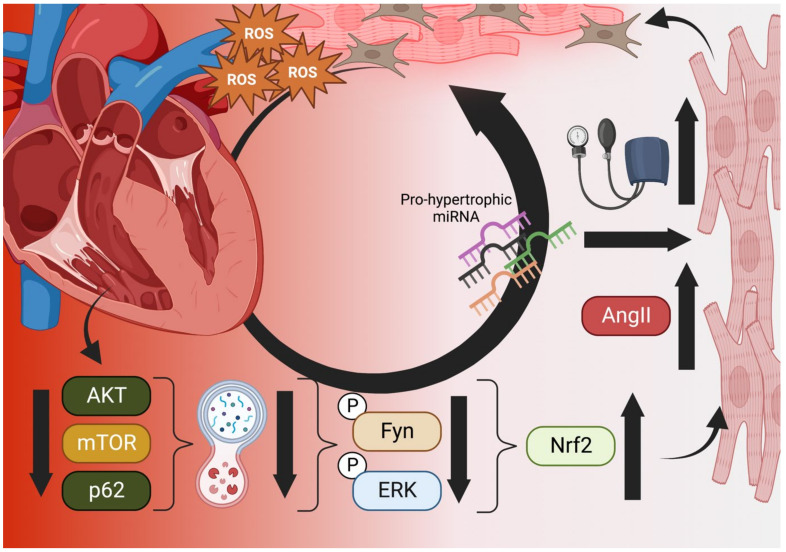
The Vicious Cycle of Nrf2 in Cardiac Hypertrophy. Aged and failing hearts have dysfunctional autophagy (**bottom**), which cannot downregulate Nrf2 transcription of Angiotensinogen and AngII, increasing blood pressure and mechanical induction of hypertrophy, pro-hypertrophic miRNA, necrosis, and fibroblast activation (**right**). Hypertrophic cells increase ROS output and decompensation within the heart occurs, increasing the ischemic microenvironment and generating even more ROS in a vicious cycle (**top**) [71,72]. Created in BioRender.com.

Of current controversy in cardiac research is the involvement of Nrf2 as a pro-hypertrophic, factor in progressive heart failure. On one side, numerous reports have linked Nrf2 deficiencies to ROS-mediated cardiac hypertrophy related to Angiotensin II, IL-6-mediated inflammation, aortic constriction (TGFβ1/SMAD2 signaling), and obesity-related stress [51,52,53]. Diverse other reports have detailed the role of Nrf2 in preventing cardiomyocyte necrosis, hypertrophy, and fibrosis of the myocardium due to ROS while antioxidant response proteins (e.g., NQO1, SOD1, GPX4) have been found at low expression levels under ischemic cardiomyopathy conditions [54,55]). However, recent evidence that Nrf2 induces progressively maladapted remodeling in the absence of functional autophagy casts doubt on the exploitation of Nrf2 in patients with metabolic disorders or heart disease (Figure 1) [46]. Reports from the Cui research group have indicated that Fyn-mediated nuclear export inhibition is to blame but other yet-discovered factors may also play crucial roles in pathogenesis [46]. Future studies on the effect of autophagy and other regulatory modalities (methylation, sumoylation, etc.) will delineate the thresholds beyond which Nrf2 enhancement becomes problematic for the heart.

Ostensibly, boosting Nrf2 will increase the total antioxidant capacity within the heart and neutralize ROS that perpetuate necrotic and fibrotic pathways, leading to the concept of “the more antioxidant capacity, the better”. In spite of this theory, results from well-controlled clinical trials of supplemental antioxidants (selenium, vitamin E, beta-carotene, etc.) have returned disappointing results where risk was either unchanged or even enhanced [56]. Results from previously reviewed meta-studies with 156,663 and 188,209 total participants found no significant effects of antioxidant/vitamin supplements on cardiovascular risk [56]). However, a recent meta-study of selenium and other antioxidants only found significant risk reduction for selenium across 43 studies (possibly because such minerals, similarly to zinc, are important constituents of antioxidant enzymes and not activators of Nrf2) [57]). Consequently, the Selenium and Vitamin E Cancer Prevention trial (N = 35,533) found that supplementation increased diabetes and prostate cancer risks, while a beta-carotene study did find inverse relationships with lower cardiovascular risk but could not completely rule out the effects of confounding variables (i.e., accidents and injuries) [57,58]. In general, antioxidants have proven to be poor substitutes for generally healthy lifestyle habits (e.g., no tobacco use, moderate diet, moderate exercise, stress reduction, good sleep habits) and excessive antioxidant use is associated with increased all-cause mortality (vitamin E), oxidative stress (ascorbic acid), and cancer risk (vitamin A) [59].

In similar fashion, Nrf2 exogenous enhancers have not shown promise in either preventing or treating cardiovascular diseases and several trials have ended early because of deleterious heart effects after treatment (see Section 4.2 and Section 4.3). For this reason, external and sustained enhancement of the antioxidant response out of context with other regulatory factors (e.g., autophagy) could counterintuitively damage the myocardium through pathways not yet fully elucidated (Figure 1). More antioxidant capacity is, in light of these studies, definitely not better.

### 3.2. Nrf2 in the Failing Heart: Autophagy as a Keystone Mechanism

Aging and failing hearts experience stiffening from fibrosis caused by immune responses to myocardial necrosis, increased ROS from aging and senescent mitochondria, lipofuscin accumulation from lysosomal degradation, deficiencies in calmodulin signaling/calcium flux (RYR2, SERCA2a) and increased maladaptive remodeling due to high blood pressure that stems from glucose dysregulation and hyperkalemia [60,61,62]. Additionally, autophagic capacity drops as suppression factors like mTOR are overexpressed by chronically high AKT levels while chronic IGF-1 expression, long touted as a youth-sustaining factor, paradoxically ages the heart rapidly as it has been shown to downregulate autophagy by suppression of autophagosome formation and increases in AKT/mTOR [63,64].

Hyperglycemia has been shown to modulate autophagy via AMPK and ROS induction of the ERK/JNK-p53 mechanism [65,66,67]. Additionally, fasting is a potent activator of autophagy even under increased peroxide generation by mitochondria in animals [66,68]. In type 2 diabetics, while initially protective, mitophagy (i.e., autophagy of damaged mitochondria) may eventually drive cells towards reduced energy as mitochondria are damaged by increased metabolic activity and are recycled faster than replacement [69]. However, the loss of autophagic capacity, especially in pancreatic *β* cells and diabetic hearts, may also be important in progression to end-stage disease [69,70]). Thus, patients who do not possess a fully intact autophagy capacity (e.g., heart failure or type 2 diabetics) may be harmed by artificial Nrf2 enhancement.

Wu et al. recently reported a putative mechanism for this effect in pressure-overloaded hearts that involves dysfunctional autophagy, restricting phosphorylated Fyn and ERK from translocating to the nucleus and downregulating Nrf2 activity that would otherwise restrict angiotensin expression [71]. In such cases, subsequent activation of angiotensin II (Ang-II) receptors by Ang-II production would increase blood pressure and eventual hypertrophy [71]. Additionally, interactions between autophagic control factor p62 and Keap1 mean that reduction in upstream p62/AKT/mTOR result in increased Nrf2 activation and further exacerbation of Ang-II-induced maladaptive remodeling (Figure 1) [72].

### 3.3. Nrf2 in the Kidneys

Because of their role in blood filtering and dependence on over ¼ of cardiac output to function, the kidneys are inextricably linked to the heart [73]. As in the heart, Nrf2 plays an important role in defense against bloodborne sources of ROS (e.g., hyperglycemia, nitrosamines, xenobiotics) and the dense, fine capillary network within kidneys is easily damaged. The primary basal ROS within the kidney are produced by epithelial cells that use mitochondrial respiration for ATP generation that drives glomerular filtration [74]. Such ROS are also important messengers in secondary pathways, including hormone secretion and vascular reactivity [73]. Transient ischemia from heart failure, atherosclerosis, or chronic kidney diseases happens from occluded blood flow and creates excessive ROS from reperfusion injury that can easily damage delicate epithelial cells within the glomerular network and release inflammatory factors that locally propagate ROS production [73]. To compensate, Nrf2, in addition to its suite of ARE-mediated antioxidant enzymes, also produces pentose phosphate that generates NADPH which serves as a local and direct antioxidant [73]. Nrf2 is also protective against heavy metal insult from cadmium or arsenic and glutathione production by Nrf2 may attenuate damage from hyperglycemia in addition to reduction in inflammation through cytokine and NLRP3 inflammasome suppression [75]). Once transcription has been activated, Nrf2 can then be degraded in its canonical manner (proteasome via β-TrCP or Hrd1) and is thus prevented from overaccumulation [75,76]).

Unfortunately, as in the heart, Nrf2 has the potential to inflict harm as a report by Rush et al. (as reviewed by Nezu and Suzuki) revealed that sustained increases of Nrf2 in injured kidneys from treatment with bardoloxone-methyl (CDDO-Me) results in proteinuria and malformed podocyte feet [76,77]. This was thought to be due to inactivation of Keap1 suppression of Nrf2 by electrophilic effect [76]. Thus, in light of the links between deficient autophagy, Nrf2, and myocardial maladaptation, similarly suppressed autophagy in aged or damaged kidneys may also modulate chronic status and permanent damage. However, as sustained autophagy after acute kidney injury has been shown to promote fibrosis, further kidney-specific studies on the relationship between autophagy, Nrf2, and kidney fibrosis with regard to acute and chronic kidney injury microenvironments are required [78].

## 4. Clinical Nrf2 Modulators

Upregulation or downregulation of Nrf2 activity by exogenous modulators can be separated into 3 groups, namely those that increase Nrf2 levels, those that facilitate Nrf2 transcription, or those that affect translocation/stability. Most electrophilic modulators derived from triterpinoids, organosulfur compounds, and stilbenes act directly on Keap1 Cys151, 273, 288 or combinations of these or other cysteines to reduce Keap1 binding affinity to cytosolic Nrf2 [79]. However, inorganic compounds, such as LiCl, may alternately activate Nrf2 through GSK-3 suppression and DHA may activate the p65/MAPK/IKK-mediated upregulation of Nrf2 [80]. By suppression of Keap1 expression, chlorogenic acid compounds (such as CGA) may also increase cytosolic free Nrf2 [81]. Other compounds, such as the fumaric acid derivatives (Tecfidera and others), increase Nrf2 activity by increasing the export of the Bach1 competitive transcription factor [82]). These compounds have been extensively reviewed [83,84]. Table 4 contains a list of currently known and reported regulators.

### 4.1. Exercise versus Exogenous Regulation

Exercise is universally accepted as heart-healthy, countering cardiomyopathy and resulting in a dramatic upregulation of Nrf2 and associated downstream elements [100]. In this fashion, Nrf2 is closely tied to muscular endurance against ROS and other associated oxidative byproducts of myocyte mitochondrial respiration. Within skeletal muscle itself, Nrf2 is part of the antioxidant response to ROS generated by aerobic respiration within striated myocytes and helps to reduce citrate synthase and COX-mediated inflammation [101]. In cases of exercise, muscle contractions (100 and 50 Hz) and long periods of aerobic exertion have been found to stimulate Nrf2 response [101]. So why does exercise-mediated Nrf2 elevation prevent cardiomyopathy instead of possibly promoting it as seen with exogenous compounds? The answer may lie in both autophagy competence and miRNA-mediated control of myocyte cell size and growth.

**Figure 2:**
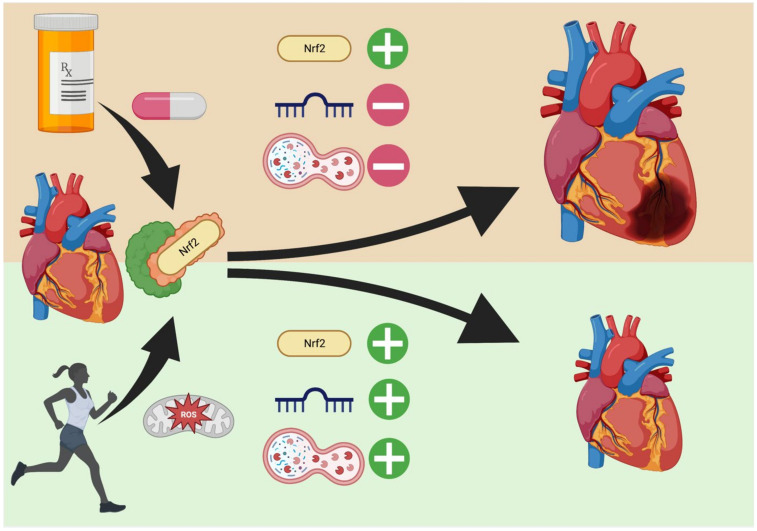
Exercise vs. Endogenous Nrf2 Regulators in the Heart. Exogenous regulators (**top**) increase Nrf2 levels through interaction with Keap1 but the effect on cardiac-related miRNA and autophagy is poorly studied. Conversely, exercise (**bottom**) upregulates not only Nrf2, but also pro- and anti-hypertrophic miRNA generation that allow for controlled remodeling. Additionally, autophagic enhancement removes the danger of necrosis from damaged organelles and reduces fibrosis from protein aggregates. Created in BioRender.com.

In the heart, murine models of exercise have reported that moderate exercise stabilizes the Nrf2 promoter in myocardial cells while it also increases AMPK phosphatase activity on mTOR to suppress its negative regulation of autophagic induction [100,102]. Additionally, it upregulates pro-autophagic factors FOXO3 and HIF-1 while also upregulating mitochondrial biogenesis factor PGC-1α through increased AMPK activity [102,103]. The simultaneous increase of autophagy with induction of short-term, shear-stress mediated pumping action, activates both SIRT1 transcription pathways as well as production of numerous anti-hypertrophic miRNAs (miR-1, -133, -26 and many others) that can, in the case of miR-1, inhibit PP2A and regulate heart rhythm or, in the case of miR-133, control hypertrophy by suppressing RHOA, NELF-A/WHSC2, and CDC42 [104,105,106,107]. Counterintuitively, pro-hypertrophic miRNAs are also expressed simultaneously (miR-143, -103, 130a, and others) that function to regulate both differentiation and cardiomyocyte morphology [108]. Furthermore, miR-29 has been specifically noted to downregulate collagen formation (reducing fibrosis) and miR-27a/b and -143 control blood pressure by action on angiotensin even as miR-27a regulates myosin heavy chain gene β-MHC (Figure 2) [105,109]. These miRNAs then act in opposing concert to mediate a controlled growth that results in myocytes that grow stronger but not larger; in effect, these cells become better adapted and more efficient. A partial list of such miRNAs affected by exercise are summarized in Table 5. In contrast, exogenous regulators of Nrf2 target only Nrf2 and do not seem to engage systemic regulatory machinery that provides anti-hypertrophic signaling in addition to the antioxidant response (Figure 2). In murine models, CDDO treatment has been shown to cause large changes in miRNA expression but this effect has not yet been studied in clinical trials of Nrf2 exogenous modulators [110]. Thus, simple administration of Nrf2 enhancers may not maintain the same benefit as exercise and clinical trials of such compounds would do well to include exercise/non-exercise groups whenever possible to determine the effect of this systemic machinery on cardiac hypertrophy. Future studies on human pan-miRNA expression profiles, especially miRNAs that regulate hypertrophy, will be useful in determining the molecular impact of artificial Nrf2 enhancement on the potential pathogenesis of cardiomyopathy.

### 4.2. Clinical Trials with Exogenous Nrf2 Modulators

To explore the potential of developing cardiac pathologies after exogenous Nrf2 modulation, ClinicalTrials.gov was searched for each compound in Table 3 and results were filtered as follows: ALL interventional trials (randomized clinical trials), any phase (Early Phase 1, Phase 1, Phase 2, Phase 3, Phase 4), with results. In cases where studies exceeded 35 (e.g., ascorbic acid), 10 of the topmost results were used. Notable cardiac-related side effects were tallied and are displayed in Table 6.

### 4.3. Clinical Trial Commentary: Reata Bardoloxone Trials

As seen in Table 4, the incidence of reported cardiac-related adverse events has remained quite low (usually less than 10%) but the number of completed trials with no results outweighs, in the cases of CDDO-Me and sulforaphane, completed trials containing reported adverse events. Even if positive results are not reported, the lack of adverse event reporting contributes to the issue of whether Nrf2 exogenous modulation has any negative effects on the heart as reported in the CDDO-Me Reata clinical trials (2007–2014; ClinicalTrials.gov: NCT01549769, NCT01351675, NCT01500798, NCT01551446, NCT01655186, NCT01576887, NCT00550849) [8]. If Nrf2 upregulation by other Keap1-Cys151-acting compounds, such as dimethyl fumarate or ursodiol (a gallstone dissolver), activated Nrf2 at the same level as CDDO, more trials could be expected to end in termination for patient safety/adverse event reasons. However, it seems as if only the Reata trials were affected because several other CDDO-Me trials were successfully completed, albeit without reported results. This raises several important questions with regard to Nrf2 regulation in chronic diseases. First, what miRNA does CDDO-Me regulate and are transcription profiles in sufferers of chronic pulmonary or metabolic diseases different from healthy volunteers? Second, since functional autophagy is indicated to play an important role in Nrf2-mediated pathogenesis, molecular evaluation of autophagy should be mandated in patients before such Nrf2-modulating compounds are tested, especially in diabetics or those with cardiac/pulmonary diseases [136]. Finally, other strong Nrf2 activators (such as organosulfur compounds or regular exercise) should be tested alongside CDDO-Me and any other compound suspected of causing Nrf2-mediated cardiomyopathy. These three precautions would give invaluable data as to the true cause of any cardiac maladaptation due to Nrf2-mediated hypertrophy and also verify miRNA-related silencing with regard to Nrf2 expression and downstream elements.

### 4.4. Clinical Perspective: Usefulness of Nrf2 Modulation in Heart Pathologies

Despite the potential to ablate ROS-mediated cardiomyocyte damage, curative applications of Nrf2 modulators for cardiac pathologies have yet to be reported. With regard to the heart, only animal models of heart failure have shown promise, with Nrf2 activators such as curcumin and CDDO-Me increasing exercise capacity, stroke volume, and cardiac output [137,138,139]). Conversely, diet and exercise carry extensive evidence for cardiac benefit. Furthermore, the possibility of exacerbating cardiomyopathy with Nrf2 exogenous modulation and concerns over chemotherapy resistance from Nrf2-mediated antioxidant enzymes upregulated in cancer cells make the use of such compounds questionable for clinical applications [47]. However, some studies have shown promise in wound healing, particularly within diabetic or hyperglycemic milieus, through the activation of Nrf2 targets HO-1 and NQO1 via hyperbaric oxygen therapy [140].

While clinical applications of Nrf2 activators have centered around cancers, kidney diseases, multiple sclerosis, and other inflammation-mediated diseases, topical Nrf2 activation in wound healing remains an underexplored topic and localized upregulation of Nrf2-mediated antioxidants might be of some value in the surgical suite [140]. Additionally, dental inflammation, primarily initiated upon microbial challenge by the NLRP3-mediated, pattern recognizing inflammasome, has been reported to be ablated by Nrf2 through NF-*κ*B downregulation, countering ROS-mediated activation [141]. Nrf2 is also apparently capable of upregulating NLRP3 upon challenge with solid irritants (e.g., alum, silica crystals) [141]. Finally, the success of Nrf2 activators in animal models naturally points towards the potential of Nrf2 manipulation in animal and veterinary medicine, especially in wound healing. Thus, the exploration of Nrf2 manipulation for human clinical purposes must orient towards localized and isolated systems (i.e., oral, digestive, neurological) instead of systemic increase through oral or intravenous administration of compounds.

## 5. Conclusions

Nrf2, once regarded as a potential key to unlock novel therapies in the cardiovascular and cancer fields, has now assumed the role of a double-edged sword: when properly regulated, it can reduce ROS and increase wound healing but carries the possibility of chemotherapy resistance and cardiomyopathy when overexpressed by exogenous manipulation through natural and synthetic compounds. Clinical trials have not reported significant effects in diverse human disease systems while only animal trials seem to hold promise for veterinary therapeutic development. However, numerous reports indicate that exercise, as a natural Nrf2 upregulator, simultaneously increases miRNA that prevent cardiomyopathy during remodeling and exercise response. Furthermore, functional autophagy prevents pathological effects of Nrf2 activation and autophagic activators, such as fasting, may also be important in controlling unwanted Nrf2 effects [66]. Therefore, detailed studies on the regulatory microenvironment of the heart during exercise, fasting, and exogenous Nrf2 stimulation may provide insight into the complex regulatory system that controls cardiac remodeling.

## Figures and Tables

**Table 1:** Neh regions of Nrf2 and their functions [9,10,11,12].

Function	Region	AA	Features
	C-Terminus		
Keap1 Binding	Neh2	16-89	Nrf2 Ubiquitin degradation domain; DLG and ETGE elements
Functional	Neh4	111-134	p300, Creb binding
Functional	Neh5	182-209	p300, Creb binding
RXRa Binding	Neh7	209-316	RXRa ARE repressor domain
Nrf2 Stabilizer	Neh6	337-394	*β*-TrCP-mediated degradation; DSGIS/DSAPGS and Ubiquitin elements
DNA Binding	Neh1	435-569	bZIP region for sMAF and ARE interaction; Cap N′ Collar region; nuclear localization
Functional	Neh3	569-605	CHD6, Creb binding
	**Carboxy** **Terminus**		

**Table 2:** Nrf2 variants associated with disease processes [7].

Variant ID/ Position	Translocation	Disease/Effect
VAR_080492/31	G > R	Immunodeficiency/developmental disorders, hypohomocysteinemia
VAR_080493/79	E > K	Immunodeficiency/developmental disorders, hypohomocysteinemia
VAR_080494/80	T > K	Immunodeficiency/developmental disorders, hypohomocysteinemia
VAR_080495/81	G > S	Immunodeficiency/developmental disorders, hypohomocysteinemia
Disease Variants		
CA16602902	E > D	Squamous and uterine cancer
CA16602899	E > Q	Lung, squamous, uterine cancers
CA16602901	E > V	Lung, squamous, uterine cancers
CA349380460	T > K	Disruption of redox homeostasis
CA349366919	E > K	Immunodeficiency/developmental disorders, hypohomocysteinemia
Mutagenic Variants	Location	Effects
Putative	79–82	Reduced Keap1 interaction
T > A	80	Reduced Keap1 interaction
E > G	82	Reduced Keap1 interaction
K > A	462	Loss of function *
K > A	472	*
K > A	487	*
R > A	499	*
R.> A	569	*

* Positions 462-569 function as a group.

**Table 4:** Exogenous Activators/Suppressors of Nrf2 [79].

Activate/Suppress	Action	Compound	Type/Origin	Ref.
**Activate**	Keap1 Cys151 alkylation	CDDO-Me	Triterpinoid	[79]
		RTA-408 (omaveloxolone)	Triterpinoid	[79]
		Oltipraz	Organosulfur	[79]
		Sulforaphane	Organosulfur	[79]
		Sulforadex	Organosulfur	[79]
		Alpha lipoic acid	Organosulfur	[79]
		ALKS-8700	Fumaric acid	[79]
		BG-12/Tecfidera	Fumaric acid	[79]
		Dimethyl fumarate	Fumaric acid	[79]
		Curcumin	Stilbene	[79,85]
		Resveratrol	Stilbene	[79]
		Ursodiol	Bile acid	[79]
		Xanthohumol	Chalcone	[86]
		Hydrogen sulfide	Inorganic	[87]
		ITH12674	Melatonin-sulforaphane	[79,88]
	Keap1 Cys 273 and 278 targeting	CXA-10	Fatty acid	[79]
	Keap1 Cys 368 and 513 targeting	CGA	Chlorogenic Acid	[89]
	Keap1 Cys 368 and 513 targeting	NMP	Chlorogenic Acid	[8]
	Inhibits phosphatase action on Nrf2	tBHQ	Quinone	[8,90]
**Activate or Suppress**	Binds to Arg415/483 or Nrf2 translocation blocker	Quercetin	Polyphenol	[8,87,91]
	Cullin3 destabilization	DHA	Lipid	[8,92]
	GSK-3 inhibition	Lithium Chloride	Inorganic	[80]
	NF-kB regulation	Melatonin	Hormone	[93]
	NR	Flazin	B-carboline alkaloid	[94]
	Competitive binding to Keap1	Apigenin	Quassinoid	[95]
**Suppress**	Nrf2 translocation blocker	Trigollenine	Alkaloid	[96]
	Nrf2 translocation blocker	Ascorbic acid	Vitamin	[97]
	Nrf2 Ub/Prot Turnover	Brusatol	Quassinoid	[98]
	Nrf2 translocation blocker	Chrysin	Quassinoid	[95]
	Nrf2 Transcriptional repressor	Luteolin	Quassinoid	[99]

**Table 5:** Hypertrophy-Modulating Factors Upregulated by Exercise [105,111].

Role	Gene/miRNA Type	Function	Ref.
Anti- Hypertrophic	miRNA-1	*HDAC4* targeting	[105,112]
	miRNA-9	Downregulation of ELAVL1-mediated inflammation	[105,113]
	miRNA-26a-1	Promotes angiogenesis; MGF*β*-SMAD2/3 signaling	[105,114]
	miRNA-98	Downregulates FAS and caspase-3	[105,115,116]
	miRNA-133	Cardiac muscle development; SFR, HDAC4, cyclin D2 targeting	[105,117]
	miRNA-145	Targets *C-MYC*, *OCT4*, *SOX2*, *KLF4*, and *STAT1*; inhibits SMC proliferation	[105,118]
	miRNA-378	Autophagy promotion via *FOXO*, *PDK1*, and *ULK1* targeting	[105,119]
Pro- Hypertrophic	miRNA-15b/107	Decreases hypoxia response; targets BCL2, ARL2, PDK4, and SGK1	[105,120,121]
	miRNA-21	Pancellular expression; Interacts with *PTEN*, *TPM1*, *PDCD4*	[105,122]
	miRNA-23a	Suppresses p53, BAX/BCL2, and PTEN	[105,123]
	miRNA-27a-3p	Targets *NOVA1* to induce hypertrophy	[105,124]
	miRNA-34	Targets *PNUTS*; enhances telomere shortening in cardiomyocytes	[105,125]
	miRNA-103	Reduces mitochondrial oxidation	[105,126]
	miRNA-143	Protein kinase C epsilon targeting	[105,127]
	miRNA-146a	Targets PLN and ANK2; reduces contractile ability	[105,128]
	miRNA-195	Targets *SIRT3*	[105,129]
	miRNA-199a -3p/5p	Controls cardiac repair via *TAOK1*, *β-TrCP*, Cofilin2	[105,130]
	miRNA-208a/b	Downregulates SOX6 and NLK	[105,131]
	miRNA-210	HIF-1alpha dependent; angiogenesis factor	[105,132]
	miRNA-221	Downregulates TGF-*β* and SMAD2, JNK1, ETS1	[105,133]
	miRNA-222	Downregulates *TGF-β* and *SMAD2*, *JNK1, ETS1*	[105,133]
	miRNA-499	Targets *p21*	[105,134,135]

**Table 6:** Selected clinical trial updates and dispositions for Nrf2 Exogenous Regulators (data from ClinicalTrials.gov).

Compound	Number of Studies	RCT ID	Year	Recruit Status	Cardiac Issues?	Notes
ALKS-8700	2	NCT02634307	2022	Complete	2/239	
		NCT03093324	2020	Complete	1/253	
Apigenin	12	NCT01286324	2017	Complete	0/17	
Ascorbic Acid	1000+	NCT03422159	2021	Complete	0/68	
		NCT03389555	2021	Complete	0/101	
		NCT03338569	2021	Complete	4/61	3/64 in placebo
		NCT00532844	2021	Complete	1/50	Combination Sapropterin DiHCl
		NCT01167569	2020	Complete	0/28	
		NCT03148236	2019	Complete	1/10	
		NCT02106975	2019	Complete	0/84	
		NCT01723696	2019	Complete	3/120	Infants
		NCT01413360	2016	Complete	0/10	
		NCT00621023	2013	Complete	1/6	
BG-12	6	NCT00273364	2020	Complete	0/55	
		NCT01568112	2016	Complete	0/42	
CDDO-Me	34	NCT04494646	2022	Complete	0/21	
		NCT03366337	2022	Complete	1/18	CDDO-Me in glomerulosclerosis
		NCT00529113	2022	Terminated	NR	Reata
		NCT02036970	2021	Complete	4/12	CDDO-me 5 mg vs. 0/4 in 20 mg
		NCT01549769	2014	Terminated	NR	Reata: Safety Concerns
		NCT01351675	2014	Terminated	NR	Reata
		NCT01500798	2014	Terminated	NR	Reata
		NCT01551446	2012	Withdrawn	NR	Reata
		NCT01655186	2012	Withdrawn	NR	Reata
		NCT01576887	2012	Withdrawn	NR	Reata
		NCT00550849	2007	Terminated	NR	Reata
Curcumin	294	NCT03085680	2022	Complete	0/8	
		NCT02494141	2022	Complete	0/34	
		NCT03584724	2022	Complete	0/20	
		NCT02978339	2020	Complete	0/15	
		NCT01383161	2020	Complete	0/25	
		NCT00094445	2020	Complete	5/44	
		NCT02104752	2019	Complete	0/17	
		NCT02300727	2019	Terminated	0/3	Not enough volunteers
		NCT01514370	2019	Complete	0/38	Plus IFNB
		NCT01740323	2019	Complete	3/15	
		NCT00641147	2017	Complete	0/21	
		NCT02556632	2017	Complete	0/64	
		NCT01246973	2016	Complete	0/344	
		NCT00365209	2015	Complete	0/22	
		NCT00525421	2013	Complete	0/10	
		NCT01042938	2012	Complete	0/14	
CXA-10	1	NCT04125745	2022	Terminated	0/1	Early termination. No safety problems
DHA	94	NCT01732874	2021	Complete	0/11	
		NCT01903525	2021	Complete	0/20	
		NCT02514070	2021	Complete	0/33	
		NCT02947100	2020	Complete	0/3	
		NCT02487771	2018	Complete	0/0	
		NCT01976806	2017	Complete	1/27	Palpitations
		NCT00266825	2016	Complete	0/154	
		NCT00100230	2015	Complete	0/27	
		NCT01007110	2014	Complete	0/35	
		NCT00440050	2014	Complete	8/214	Pulmonary embolus
Dimethyl Fumarate	131	NCT02981082	2022	Terminated	0/4	Low recruitment
		NCT04570670	2022	Complete	1/50	
		NCT02739542	2022	Complete	0/44	
		NCT02634307	2022	Complete	1/225	ALKS-8700 2/239
		NCT02907177	2021	Terminated	1/68	Low recruitment
		NCT02975349	2021	Active no recruit	0/54	
		NCT00835770	2020	Complete	9/868	3x/day 11/868 2x/day
		NCT03093324	2020	Complete	0/251	ALKS-8700 1/253
		NCT02428231	2020	Terminated	0/32	
		NCT02410278	2020	Complete	0/98	
		NCT02951533	2020	Complete	0/84	
		NCT03331835	2020	Complete	0/102	
		NCT00273364	2020	Complete	0/55	
		NCT02784834	2019	Terminated	0/2	No funding
		NCT02525874	2019	Complete	1/218	
		NCT02555215	2019	Complete	1/20	
		NCT03255382	2019	Complete	0/57	Fumaderm
		NCT02634801	2019	Complete	0/19	
		NCT02438137	2017	Complete	1/21	MI
		NCT02125604	2017	Complete	0/211	
		NCT01156311	2017	Complete	3/57	With IFNB, 1/47 with Glatiramer acetate
		NCT02430532	2017	Terminated	0/28	Sponsor terminated
		NCT02410200	2017	Complete	0/22	
		NCT01873417	2017	Complete	1/233	
		NCT02343159	2017	Terminated	1/27	Angina; Sponsor Decision
		NCT02097849	2017	Complete	0/38	
		NCT02117050	2017	Terminated	0/0	Low recruitment
		NCT02217982	2017	Terminated	0/4	Low recruitment
		NCT02474082	2017	Complete	0/95	
		NCT02241785	2017	Terminated	0/47	Business Reasons
		NCT02090413	2016	Complete	0/80	
		NCT01568112	2016	Complete	0/43	
		NCT00420212	2015	Complete	3/826	
		NCT00451451	2015	Complete	1/703	
Melatonin	49	NCT02654314	2022	Terminated	0/136	
		NCT02386319	2021	Complete	0/16	
		NCT03597529	2021	Complete	0/40	
		NCT02344316	2021	Complete	0/13	
		NCT02631148	2021	Terminated	0/12	
		NCT04137627	2019	Complete	0/13	
		NCT01700959	2018	Complete	9/66	delayed sleep onset
		NCT00925899	2017	Complete	0/34	
		NCT01805089	2015	Complete	0/48	
		NCT01355523	2014	Terminated	1/27	Inclusion/finances
Quercetin	109	NCT02195232	2021	Complete	0/57	
		NCT02463357	2021	Complete	0/20	
		NCT01722669	2020	Complete	0/30	
		NCT01708278	2016	Complete	0/6	
		NCT00913081	2015	Complete	0/17	
Resveratrol	192	NCT01354977	2022	Complete	0/12	
		NCT02523274	2022	Complete	0/20	
		NCT04668274	2022	Complete	0/24	
		NCT01321151	2021	Complete	0/6	
		NCT02767869	2021	Complete	0/12	
		NCT02114892	2020	Complete	0/12	
		NCT03384329	2020	Complete	0/11	
		NCT00920556	2019	Terminated	2/24	At study directors discretion
		NCT02095873	2017	Complete	0/32	
		NCT02475564	2016	Complete	0/22	
		NCT01504854	2016	Complete	1/64	
		NCT01640197	2012	Complete	0/30	
RTA-408	2	NCT02259231	2021	Complete	5/41	
		NCT02255422	2021	Complete	3/40	
Sulforaphane	92	NCT03126539	2022	Terminated	0/9	Adminstrative
		NCT03402230	2022	Complete	1/49	
		NCT02810964	2021	Complete	1/32	Placebo 3/32
		NCT02561481	2020	Complete	0/32	
		NCT02909959	2020	Complete	0/24	
		NCT02885025	2020	Complete	0/30	
		NCT00621309	2019	Complete	0/29	
		NCT02656420	2019	Complete	0/115	
		NCT01474993	2018	Complete	0/26	
		NCT00982319	2018	Complete	0/15	
		NCT01437501	2018	Complete	0/148	
		NCT01228084	2017	Complete	0/20	
		NCT01335971	2017	Complete	1/29	COPD exacerbation
		NCT00843167	2017	Complete	0/27	
		NCT01845220	2017	Complete	0/30	
		NCT00946309	2016	Complete	0/21	
Ursodiol	182	NCT02748616	2022	Complete	0/2	
		NCT03226067	2022	Complete	0/74	
		NCT03602560	2022	Complete	0/188	
		NCT02955602	2022	Complete	2/108	
		NCT01865812	2022	Complete	0/26	
		NCT03633227	2022	Terminated	2/10	Sponsor decision
		NCT00059202	2021	Terminated	0/76	Futility
		NCT03394924	2021	Complete	0/59	
		NCT02078882	2020	Complete	1/16	
		NCT00706381	2020	Complete	0/29	
		NCT03742973	2020	Terminated	0/0	Low recruitment
		NCT04053023	2020	Complete	0/19	
		NCT02033876	2019	Complete	0/12	
		NCT01904058	2019	Complete	1/20	
		NCT03124108	2019	Complete	0/30	
		NCT02244944	2018	Terminated	0/2	Low recruitment
		NCT00575042	2018	Complete	0/20	
		NCT01097304	2017	Complete	6/36	
		NCT01389973	2016	Complete	0/20	
		NCT02111603	2016	Complete	0/12	
		NCT00877604	2014	Complete	0/15	
		NCT01249092	2013	Complete	0/20	
		NCT00200343	2012	Complete	0/596	
		NCT00550862	2012	Complete	1/41	
		NCT00909753	2009	Complete	NR	
		NCT00909610	2009	Complete	NR	
